# The Ventricular System Enlarges Abnormally in the Seventies, Earlier in Men, and First in the Frontal Horn: A Study Based on More Than 3,000 Scans

**DOI:** 10.3389/fnagi.2019.00294

**Published:** 2019-11-05

**Authors:** Antonio Currà, Francesco Pierelli, Riccardo Gasbarrone, Daniela Mannarelli, Italo Nofroni, Vittoria Matone, Lucio Marinelli, Carlo Trompetto, Francesco Fattapposta, Paolo Missori

**Affiliations:** ^1^Academic Neurology Unit, A. Fiorini Hospital, Terracina, (LT), Department of Medico-Surgical Sciences and Biotechnologies, Sapienza University of Rome—Polo Pontino, Rome, Italy; ^2^Academic Neuro-Rehabilitation Unit, ICOT, Latina, IRCCS Neuromed and Department of Medical-Surgical Sciences and Biotechnologies, Sapienza University of Rome—Polo Pontino, Rome, Italy; ^3^Department of Chemical Engineering, Materials & Environment, Sapienza University of Rome, Rome, Italy; ^4^Neurology Unit, Department of Human Neurosciences, Policlinico Umberto I, Sapienza University of Rome, Rome, Italy; ^5^Department of Public Health and Infectious Diseases, Medical Statistics and Biometry, Sapienza University of Rome, Rome, Italy; ^6^Neurosurgery Unit, Department of Human Neurosciences, Policlinico Umberto I, Sapienza University of Rome, Rome, Italy; ^7^Department of Neuroscience, Rehabilitation, Ophthalmology, Genetics, Maternal and Child Health, University of Genova, Genova, Italy; ^8^Department of Neuroscience, IRCCS Ospedale Policlinico San Martino, Genova, Italy

**Keywords:** aging, brain, enlargement, Evans’ index, hydrocephalus, normal pressure, ventricular system

## Abstract

**Objectives**: To detect on computed tomography (CT) brain scans the trajectories of normal and abnormal ventricular enlargement during aging.

**Methods**: For each 1-year age cohort, we assessed in 3,193 axial CT scans the Evans’ index (EI) in the anterior frontal horns and the parieto-occipital (POR) and temporal ratio (TR) in the posterior and inferior horns. Cut-off values for abnormal enlargement were based on previous clinical studies.

**Results**: The mean age associated with normal linear measures was 71 years. Values for all three measures increased with age, showing a linear relationship below—but not above—each cut-off value. The mean age of participants with abnormal enlargement on CT progressed from 79 years for EI to 83 years for POR to 87 years for TR. These results suggested that ventricular dilatation progresses in an age–location relationship. First comes enlargement of the frontal horns (13.8% of scans), followed by the parieto-occipital horns (15.1% of scans) and then temporal horn enlargement (6.8% of scans). Scans from men displayed abnormal values earlier than scans from women (on average 6 years). Risk increased 5.1% annually for abnormal EI, 9.0% for abnormal POR, and 11% for abnormal TR (all *p* < 0.001). The most frequent agreement between categories (normal–abnormal) for values of neuroimaging measures was identified for POR–TR.

**Conclusion**: The results of this large radiological study suggest that the ventricular system enlarges progressively during aging, and in a subset of patients follows an abnormal consecutive geometric dilatation, influenced by age and sex.

## Introduction

The cranial cavity contains brain, vessels, and cerebrospinal fluid (CSF), largely in the cerebral ventricular system. With normal aging, the relative volumes of these components may change, predominantly those of brain and CSF, given that blood supply can be considered basically invariant.

Brain and cranial CSF volumes can be evaluated with standard neuroimaging techniques [i.e., computer tomography (CT) and magnetic resonance imaging, MRI], which may show brain atrophy (i.e., loss of brain volume), ventricular enlargement with secondary increase in ventricular CSF, or both. Variable combinations of such neuroimaging findings are seen in a number of neurodegenerative, vascular, and demyelinating diseases, but ventricular enlargement in isolation can be observed specifically in idiopathic chronic hydrocephalus. The Evans’ index (EI; Evans, 1942) allows for indirect linear measurement of ventricular size on standard CT and MRI. EI values >0.30 are considered indicative of pathological ventricular enlargement (i.e., hydrocephalus; Inatomi et al., 2008; Ambarki et al., 2010).

The progression of ventricular enlargement can be monitored with serial neuroimaging both in symptomatic and healthy elderly individuals (Missori and Curra, 2015). Indeed, during aging, the cerebral ventricles expand (Missori et al., 2016). Unfortunately, using EI as a surrogate marker of ventricular volume in adults (Earnest et al., 1979; Ambarki et al., 2010) prevents the determination of whether pathological volume enlargement relates to brain atrophy or disturbance in CSF. In addition, when EI is measured in scans from normal elderly people, abnormally high EI values are found in 2.8%–17% of scans (Inatomi et al., 2008; Ambarki et al., 2010) and this percentage increases when scans are consecutively recruited from an emergency department (ED; Missori et al., 2016). Therefore, EI alone has a limited role in predicting whether ventricular enlargement fosters a neurological condition, even after the recent re-visitation of the cut-off values (Brix et al., 2017).

By examining two other linear indexes of ventricular volume [the parieto-occipital ratio (POR) and the temporal ratio (TR)] in addition to EI, we showed that during aging, the ventricular system enlarges progressively but non-uniformly, leading to disproportionate parieto-occipital and temporal dilatation in patients with neurological impairment compared with asymptomatic adults (Missori and Curra, 2015). The neurological signs/symptoms found in diseases that manifest with ventricular enlargement include cognitive impairment, gait disturbances, and urinary sphincter abnormalities. A small part of the spectrum of these signs/symptoms has some overlap with subtle changes in cognition, gait, and micturition frequently observed in otherwise “normal” elderly people (e.g., generic psychomotor slowing, difficulty in multistep procedures or formulation of abstractions, erratic impairment of recall of recent events, difficulty in tandem gait, changes in step length and height or in body sway during walking, occasional urinary urgency). Having readily applicable and simple neuroimaging measures that help predict which asymptomatic adults are on a trajectory to develop neurological diseases would be useful in clinical practice because it would prompt early monitoring, and if possible, eventual prevention.

For this reason, we designed a transversal radiological study to assess changes in ventricular volume with age, independent of eventually associated neurological conditions. We analyzed a larger series (>3,000) of consecutive head scans from an ED, evaluating the proportion of pathological values for each measure (EI, POR, and TR) in 1-year age cohorts, the proportion of two or more measures altered, and the relative proportion of abnormality between measures.

## Materials and Methods

We examined head CT data from individuals (aged 45–103 years) admitted to our ED between June 2009 and October 2010. All scans were part of an early work-up for acute neurological symptoms or head injury. We excluded from analysis all scans showing hemorrhagic strokes, neoplasms, or post-traumatic hemorrhages.

**Figure 1:**
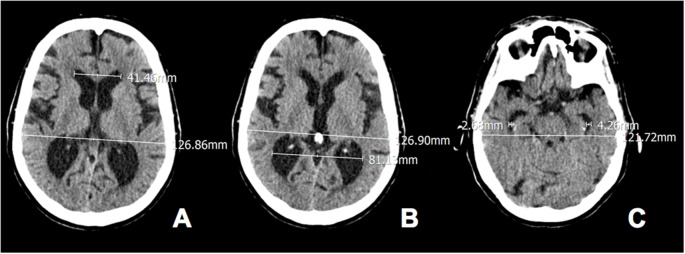
Calculated Evan’s index, parieto-occipital ratio (POR) and temporal ratio (TR) in axial computed tomography (CT) scans of a 80-year old woman. **(A)** Evans’ index (EI) 41.46/126.86 = 0.33; **(B)** POR 81.13/126.90 = 0.64 and **(C)** TR 2.68 + 4.26/121.72 = 0.06.

We calculated EI in the axial scans by dividing the width of the frontal horns at the level of Monro’s foramens (i.e., a reliable anatomical neuroimaging landmark to make the calculation procedure unequivocal) by the maximum width of the inner table of the cranium (Figure 1; Missori and Curra, 2015). According to the guidelines for normal pressure hydrocephalus, EI values >0.30 were considered to be pathological. The POR was calculated as the ratio between the width of the occipital horns at the atrium and the maximum width of the inner table of the cranium at the same level. The TR was calculated as the ratio between the width of the temporal horns at the level of maximal convexity of the hippocampus and the maximum width of the inner table of the cranium at the same level. Because in our previous series (Missori and Curra, 2015) cognitively impaired patients all showed POR > 0.55 and TR > 0.07, here we chose these values as the cut-offs for identifying abnormal parieto-occipital and temporal enlargement.

Two observers (one neuroradiologist, one neurosurgeon) calculated the three measures in the axial scans. Agreement between observers was evaluated using kappa statistics.

Student’s *t*-test, chi-squared test, and regression analysis (multiple and linear) of EI, POR, and TR values were obtained from all scans, 1-year (single-age) cohorts, and sexes. Statistical significance was set at *p* < 0.01.

## Results

Out of 3,450 examined CT scans, 3,193 were eligible for EI calculation, representing the starting point of visual analysis of scans to categorize the ventricles as “enlarged” or “normal.” A kappa value of 0.86 indicated “*almost perfect agreement*” between observers in ventricles’ categorization by using the radiological measure EI. Agreement in categorizing ventricles as “enlarged” or “normal” by using the measures POR and TR provided Kappa values of 0.85 and 0.83, respectively.

The eligible scans came from 1,711 women [53.6%, age (mean ± SD) 74.7 ± 14.2 years] and 1,482 men (46.4%, age 70.3 ± 13.2 years; entire study population, age 72.7 ± 13.9 years). EI values were normal (i.e., ≤0.3) in 2,752 scans (86.2%, EI 0.26 ± 0.03, age 71.6 ± 13.9 years); POR values were normal (i.e., ≤0.55) in 2,713 scans (84.9%, POR 0.50 ± 0.03; age 70.8 ± 13.7 years); and TR values were normal (i.e., ≤0.07) in 2,977 scans (93.2%, TR 0.03 ± 0.02; age 71.7 ± 13.6 years).

Table 1 shows the descriptive statistics for all neuroimaging measures. The values for EI and POR were significantly greater in men than in women (*p* < 0.001), whereas TR was similar between sexes (*p* < 0.37; for all comparisons Student’s *t*-test). The mean values for all linear measures were in the normal range.

**Table 1:** Values of neuroimaging linear measures.

		EI	POR	TR	Age (years)
All	mean	0.27	0.51	0.03	72.6
	SD	0.03	0.05	0.03	13.9
	*n*	3,193	3,193	3,193	3,193
	min	0.11	0.29	0.01	45.0
	max	0.61	0.68	0.21	104.0
Women	mean	0.27	0.51	0.03	74.7
	SD	0.04	0.05	0.03	14.2
	*n*	1,712	1,712	1,712	1,712
	min	0.13	0.29	0.01	45.0
	max	0.61	0.68	0.21	104.0
Men	mean	0.28	0.52	0.03	70.3
	SD	0.03	0.04	0.03	13.2
	*n*	1,481	1,481	1,481	1,481
	min	0.11	0.39	0.01	45.0
	max	0.45	0.68	0.20	101.0

The abnormal values for the neuroimaging measures are shown in Table 2. Abnormal values for all measures were similar between sexes. Men exhibited abnormal values earlier than women (*p* < 0.01, Student’s *t*-test), and abnormal values for EI emerged earlier than for POR, which in turn emerged earlier than values for TR (for all comparisons *p* < 0.01).

**Table 2:** Abnormal values (aValues) of neuroimaging linear measures.

		EI	POR	TR
All	aValues (*n*)	440	482	217
	aValue	0.33 ± 0.03	0.58 ± 0.02	0.11 ± 0.03
	% abnormal	13.8	15.1	6.8
	age	79.3 ± 11.8	83.3 ± 10.2	86.5 ± 9.9
Women	aValues (*n*)	184	230	121
	aValue	0.33 ± 0.03	0.58 ± 0.02	0.11 ± 0.03
	% abnormal	5.8	7.2	3.8
	age	82.9 ± 11.2	86.4 ± 9.4	89.3 ± 9.8
Men	aValues (*n*)	256	252	96
	aValue	0.33 ± 0.02	0.58 ± 0.02	0.11 ± 0.03
	% abnormal	8.0	7.9	3.0
	age	76.8 ± 11.7	80.5 ± 9.9	82.9 ± 8.9

**Figure 2:**
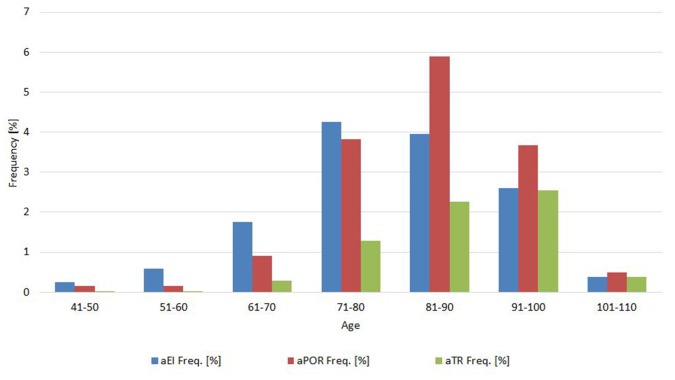
Proportion of abnormal values for each measure in 10-year age cohorts.

Frequency analysis showed that 2,457 scans (76.9%) all had normal neuroimaging measures, 436 scans (13.7%) had one abnormal measure, 199 scans (6.2%) had two abnormal measures, and 101 (3.2%) had all abnormal neuroimaging measures. The relative frequency of abnormal values for each measure changed according the age (see 10-year cohorts in Figure 2).

**Figure 3:**
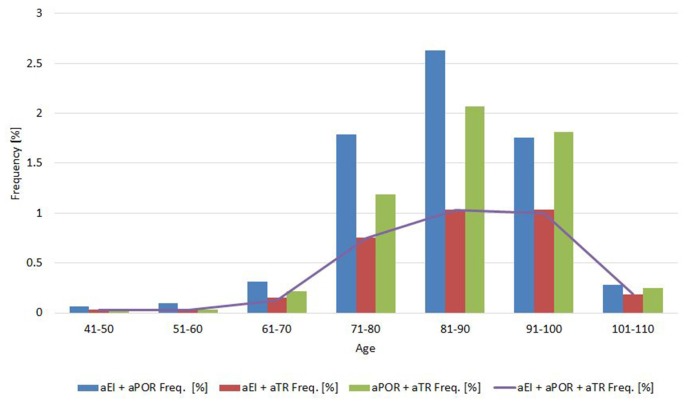
Proportion of abnormal values for pairs of (and all) neuroimaging measures in 10-year age cohorts.

Concomitance, or how frequently two measures were both normal or both abnormal, was 39% for EI and POR (normal/abnormal, EI-POR and aEI-aPOR; Cohen’s kappa, 0.39 ± 0.045); 25% for EI and TR (Cohen’s kappa, 0.245 ± 0.047); and 46% for POR and TR (Cohen’s kappa, 0.46 ± 0.047; *p* < 0.0001). When the same analysis was made for sex, the concomitance between categories of EI and POR was 34% in men (Cohen’s kappa, 0.338 ± 0.06) and 45% in women (Cohen’s kappa, 0.449 ± 0.035). Between categories of EI and TR, it was 21% in men (Cohen’s kappa, 0.21 ± 0.063) and 29% in women (Coen *K* = 0.288 ± 0.072). Concomitance between categories of POR and TR was 46% in men (Cohen’s kappa, 0.46 ± 0.065), and 46% in women (Cohen’s kappa, 0.46 ± 0.067). The frequency of concomitance for abnormal values also changed with age (Figure 3).

**Figure 4:**
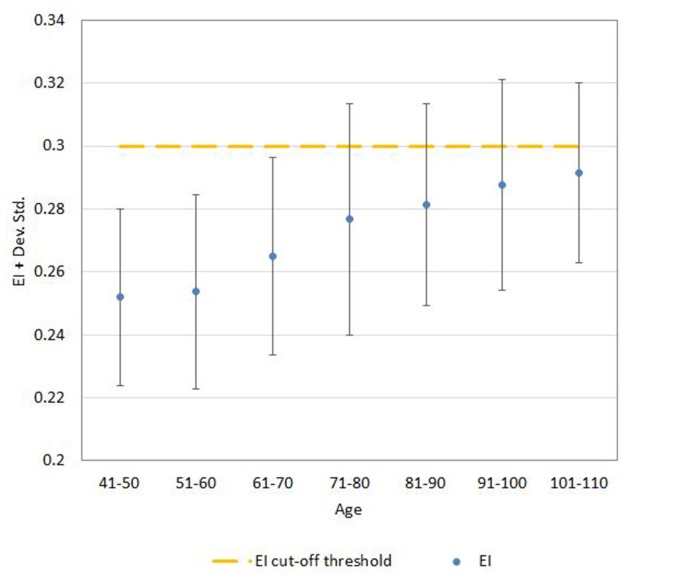
Mean ± SD values for EI neuroimaging measure in each age (10-year) cohorts. Note: the upper part of the bar indicates the 97.5 percentile.

**Figure 5:**
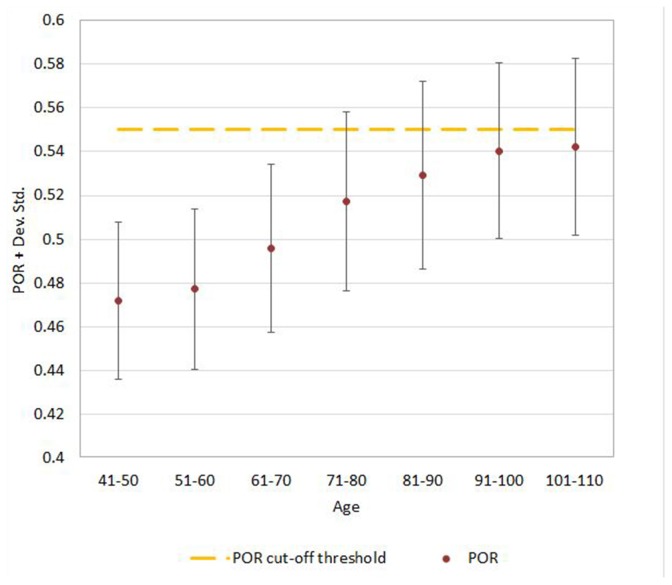
Mean ± SD values for POR neuroimaging measure in each age (10-year) cohorts. Note: the upper part of the bar indicates the 97.5 percentile.

**Figure 6:**
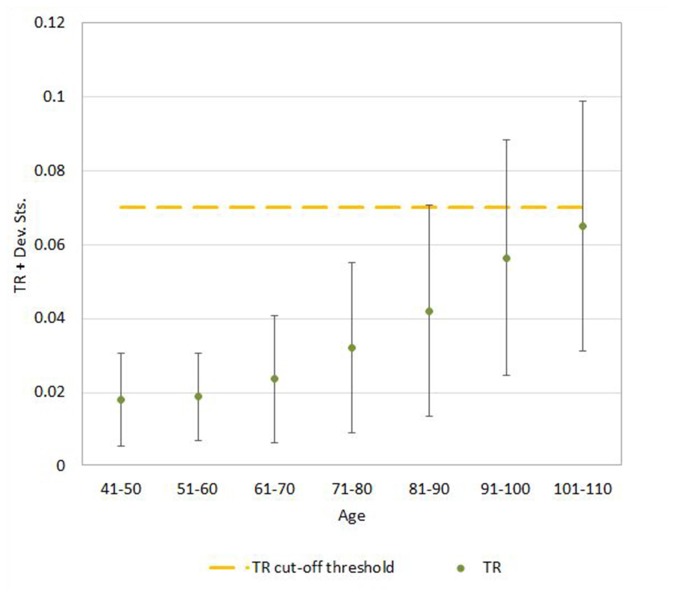
Mean ± SD values for TR neuroimaging measure in each age (10-year) cohorts. Note: the upper part of the bar indicates the 97.5 percentile.

Regression analysis demonstrated that the risk for having abnormal neuroimaging measures depended on age (Table 3 and Figures 4, 5, 6) and sex. The model of simple linear regression with age fit better for POR and TR in comparison to EI, and for values of neuroimaging markers below the cut-off for abnormality.

**Table 3:** Summary of regression analysis.

	*X* = Age, *Y* = EI	*X* = Age, *Y* = EI ≤ 0.3	*X* = Age, *Y* = EI > 0.3	Women *X* = Age, *Y* = EI	Men *X* = Age, *Y* = EI
*R^2^*	0.113	0.0962	0.005	0.128	0.136
*ρ*	0.336	0.310	0.071	0.358	0.369
*RMSE*	0.032 EI	0.025 EI	0.025 EI	0.033 EI	0.031 EI
*Fitted vs. residual*	linear	pseudo linear	not linear	linear	linear
	***X* = Age, *Y* = POR**	***X* = Age, *Y* = POR ≤ 0.55**	***X* = Age, *Y* = POR > 0.55**	**Women *X* = Age, *Y* = POR**	**Men *X* = Age, *Y* = POR**
*R^2^*	0.241	0.188	0.0011	0.277	0.258
ρ	0.491	0.434	0.033	0.526	0.508
*RMSE*	0.039 POR	0.031 POR	0.024 POR	0.039	0.038
*Fitted vs. residual*	linear	pseudo linear	not linear	linear	linear
	***X* = Age, *Y* = TR**	***X* = Age, *Y* = TR ≤ 0.07**	***X* = Age, *Y* = TR > 0.07**	**Women *X* = Age, *Y* = TR**	**Men *X* = Age, *Y* = TR**
*R^2^*	0.213	0.229	0.007	0.232	0.208
*ρ*	0.462	0.479	0.084	0.482	0.456
*RMSE*	0.023 TR	0.013 TR	0.028 TR	0.023 TR	0.022 TR
*Fitted vs. residual*	not linear	not linear	not linear	not linear	not linear

The risk for having abnormal EI increased 5.1% per year, the risk for having abnormal POR increased 9.0% per year, and that for abnormal TR increased 11% per year (all, *p* < 0.001). Men had greater risk than women for having abnormal EI (128%), aPOR (114%), and abnormal TR (51%; all *p* < 0.01).

## Discussion

This transversal neuroimaging study of >3,000 consecutive ED head CT scans provides evidence that the linear measure of ventricular volume that is most frequently found to be abnormal is POR (15.1%), followed by EI (13.8%), and then by TR (6.8%). For each linear measure, the mean age of participants with scans exhibiting normal values was 71 years, but for scans exhibiting abnormal values, the mean age was 79 years for EI, 83 years for POR, and 87 years for TR. Scans from men displayed abnormal values earlier than scans from women (on average 6 years), but the time intervals of age progression to abnormal values were similar between the sexes. Values for EI and POR were greater in men than in women. Among scans displaying abnormal values, 60% had only one abnormal measure, 27% had two, and 13% had all abnormal measures. The most frequent concomitance between the categories (normal/abnormal) of values for the neuroimaging measures was found for POR–TR, followed by EI–POR, and then EI–TR. Regression analysis showed that age confers unequal risk per year for developing abnormal values for the three measures (TR > POR > EI). Sex influenced values for POR and EI, with higher and more frequently abnormal values in men than in women. In addition, sex changed the proportions of concomitance between EI–TR and EI–POR, but not between POR–TR. Values for all three measures increased with age, showing a linear relationship below but not above each cut-off value.

The first point to discuss is the difference between the 13.8% value for abnormal EI we found in this series compared with the 19.3% previously reported in a smaller but analogous series (Missori et al., 2016). In the current work, in seeking to be unequivocal, to calculate EI, we measured the width of the frontal horns in the lower axial CT slice at the level of Monro’s foramen, which we considered a reliable anatomical neuroimaging landmark. At this level, the width of the frontal horns is shorter than that measured in the CT slice immediately following. Our calculation procedure was, therefore, more specific in detecting abnormal values because of the lower values at the numerator of the ratio, and reasonably is clinically more stringent. Overall, the percentage of aEI identified with the procedure adopted is in line with previous studies (Earnest et al., 1979; Inatomi et al., 2008; Ambarki et al., 2010).

Rates of abnormal values appear to be highest in the 70–90 year-old groups, but tended to reduce in the “oldest” old groups (>90). Various factors may have determined the result. First, the lower numerosity of the “oldest” age classes compared to the “middle elderly” classes. Second, a possible difference in the accessibility to ED department in the distinct age classes. Finally, we cannot exclude that survivors in the “oldest” classes may be “selected” by many positive factors, including near-normal morphology of the cerebral ventricular system.

We found that the most frequent abnormal linear measure of ventricular enlargement in the scans was POR, followed by EI, and then TR. The corresponding mean age values indicated that enlargement of the frontal horns develops first, followed by the most common occurrence, enlargement of the parieto-occipital horns, and then the least frequent development, enlargement of the temporal horns. Thus, in most individuals, the frontal horns begin to dilate first, followed by the parieto-occipital, and finally the temporal horns. Similar to blood vessels and other closed systems in the body (i.e., heart, bladder, uterus), such a spatial progression could be explained by Pascal’s principle (same pressure in all regions of the ventricular system) and LaPlace’s law (greater wall tension for greater radius of the cavity). Therefore, the ventricle horn enlargement progresses from frontal to parieto-occipital and then temporal horns because the length of the radius in these horns reduces accordingly, changing the wall tension required to withstand the given internal fluid pressure.

In the present study, the frequency of agreement between values for the neuroimaging measures was analyzed and found to differ between measures. Concomitance indicates which proportion of the values assumed by two different measures belongs to the same category, or how frequently the two are either both normal or both abnormal. Clinically, concomitance suggests that the size of two distinct parts of the ventricular system undergoes the same natural history, i.e., are subject to similar factors over time, and respond to them in a similar manner. The greatest proportion of concomitance we found is for values for POR and TR (almost 50%), suggesting that the strongest links between sizes of distinct parts of the ventricular system are for parieto-temporal horns. Concomitance for abnormal POR–abnormal TR would reasonably reflect neurological conditions leading to loss of brain volume in the parieto-temporo-occipital carrefour, which becomes increasingly frequent with age (Ten Kate et al., 2018). Concomitance between categories of EI and POR indicates that 60% of scans show EI and POR in opposite categories. We interpret this finding as suggesting that the enlargement in the frontal and parieto-occipital horns usually takes place one after the other, as supported by the lower mean age of participants with abnormal EI than those with abnormal POR. As has been suggested, frontal to parieto-occipital progression could be related to the physics of CSF circulation (Bradley, 2015) the anatomical structure of the ventricular system organized as sequential cavities, and the mini-gradients of CSF pressure and volumes triggered by physiological or pathological conditions that alter CSF absorption (Missori and Curra, 2015).

Of note, there is an important distinction between the cut-off values for POR and TR and for EI. On the one hand, POR and TR cut-off values have been selected on clinical grounds, i.e., the absence of cognitive impairment or gait or urinary abnormalities in patients with measures below the cut-off (Missori and Curra, 2015). On the other hand, EI was designed as a pure radiological measure independent from clinical features (Evans, 1942). This difference implies that overlapping abnormal EI values can be found both in normal elderly people and in patients with neurological conditions, whereas abnormal POR and abnormal TR values can be identified—by definition—only “in patients.” This point is decisive when interpreting the abnormal EI–abnormal POR and abnormal EI–abnormal TR concomitance, because the implication is that none of the “concomitant abnormal EIs” can be found in normal elderly individuals. In our opinion, “concomitant abnormal EIs” may be regarded as a better-suited method than EI-only for suspecting a neurological disease on the sole basis of brain scans. Brain scans with two or more concomitant abnormal values reasonably reflect neurological conditions leading to loss of brain volume or disturbance in CSF dynamics. Although not all brain volume changes portend cognitive impairment, we consider the presence of concomitant abnormal EI should prompt a search for neurological conditions. Supporting this conclusion, we provide a small but meaningful sample of clinical data evaluating cognitive, gait, and urinary function in 11 out of 3,193 subjects whose scans were examined in this large-scale series. These 11 subjects were followed both clinically and radiologically for at least 4 years (Missori and Curra, 2015) and their most recent imaging markers are part of the present study dataset. Clinical data along with imaging markers values are displayed in Table 4, showing that the nine subjects having “concomitant abnormal EIs” (i.e., subjects 1–4, 6–8, 10–11), all exhibit various combinations of cognitive, gait, and urinary dysfunction.

**Table 4:** Patients’ clinical evaluation and radiological indexes of a sample of 11 out 3,193 scans.

Subject	Cognitive	Gait	Urinary	EI	POR	TR
1	++	++	++	0.33	0.61	0.16
2	+++	+	++	0.35	0.57	0.21
3	+++	++	++	0.35	0.64	0.3
4	++	++	+	0.38	0.55	0.16
5	+++	+++	++	0.28	0.58	0.22
6	+++	+++	++	0.36	0.67	0.17
7	+++	++	++	0.40	0.67	0.13
8	+++	+++	++	0.38	0.63	0.13
9	++	++	++	0.30	0.65	0.10
10	++	++	+	0.32	0.58	0.15
11	++	+++	++	0.33	0.60	0.07

*Cognitive impairment was graded according to mini-mental state examination (MMSE) score as severe (+++ score <10 points), moderate (++ score between 10–19 points), or mild (+ score 20–24 between points). Gait impairment was evaluated as unsteady (+); walking only with help (++); bedridden (+++). Urinary impairment was graded as urgent micturition (+) or urinary incontinence (++)*.

We found that values for all three neuroimaging measures increased with age. This finding confirms previous studies that the ventricular volume increases slowly but unavoidably as people grow older (Earnest et al., 1979; Laffey et al., 1984; Stafford et al., 1988). Of interest, the present series of scans showed that the relationship between age and ventricular size is not linear for values over the cut-off for each measure of ventricular enlargement, suggesting that the rate of ventricular enlargement changes after the onset of multidomain cognitive impairment, as shown previously (DeCarli et al., 1992). Reasonably, this means that normal aging causes a slow and low ventricular enlargement with linear progression, whereas neurologic conditions do not. Because the linear relationship between age and value differs for EI vs. POR and EI vs. TR, we speculate that age confers unequal risk per year for developing ventricular enlargement in the different horns. The effect of normal aging on ventricular volume is not uniform, being greatest for the temporal horns and lowest for the frontal horns. This pattern is consistent with reported longitudinal findings (Raz et al., 2005) indicating that the hippocampus—the main brain structure protruding into the temporal horn—is far from being spared in normal aging (Walhovd et al., 2011).

Sex influences POR and EI values, which are greater and more frequently abnormal in men than in women. This finding has been reported in previous studies of healthy elderly people and of patients recruited for a large dementia database (Walhovd et al., 2011; Brix et al., 2017) but it contrasts with other studies showing that ventricular volumes corrected for differences in cranial size do not vary between sexes (Cosgrove et al., 2007; Fjell et al., 2009). However, the role of sex hormones in the brain is well known, and the thickening in cortical regions is associated with changes in testosterone levels (Zubiaurre-Elorza et al., 2014). Also, sex-specific differences in dopaminergic, serotonergic, and γ-aminobutyric acid-ergic markers indicate that male and female brains are neurochemically distinct (Cosgrove et al., 2007). By examining abnormal values alone, we found that sex affects the proportion of scans but not the dimension of ventricular size. We interpret this finding as indicating that ventricular volume changes disproportionately in men and women with increasing age, but in approximately the same way. Also, we found that sex influences the proportions of concomitance EI–TR and EI–POR (but not POR–TR). Therefore, we conclude that the role that sex plays in ventricular enlargement is complex and probably multidimensional.

The advantages of our study include the large number of scans, ease of measurement, and high inter-observer reliability in the neuroimaging measurements. Disadvantages include the lack of clinical information about participant health and cognition (although this was not an explicit aim of the study), and knowledge that measuring ventricular volume is the best strategy for diagnosing ventricular enlargement. However, advanced neuroimaging techniques for ventricular volume measurement are time-consuming, require specialized software and expertise, and are not readily available in most resource-limited settings, thus proving unhelpful for routine quantitation of the ventricular size.

## Data Availability Statement

The datasets generated for this study are available on request to the corresponding author.

## Ethics Statement

Ethical review and approval was not required for the study on human participants in accordance with the local legislation and institutional requirements. Written informed consent for participation was not required for this study in accordance with the national legislation and the institutional requirements.

## Author Contributions

All authors have made substantial contributions as detailed in the following text. AC, FP, RG, DM, IN, VM and PM: the conception and design of the study, or acquisition of data, or analysis and interpretation of data. AC, FP, RG, DM, IN, LM, CT, FF and PM: drafting the article or revising it critically for important intellectual content. AC, FP, RG, DM, VM, LM, CT, FF and PM: final approval of the version to be submitted.

## Conflict of Interest

The authors declare that the research was conducted in the absence of any commercial or financial relationships that could be construed as a potential conflict of interest.
